# Effect of PGE_2_-EP_s_ pathway on primary cultured rat neuron injury caused by aluminum

**DOI:** 10.18632/oncotarget.21122

**Published:** 2017-09-21

**Authors:** Lu Yang, Yuling Wei, Ying Luo, Qunfang Yang, Huan Li, Congli Hu, Yang Yang, Junqing Yang

**Affiliations:** ^1^ Department of Pharmacology, Chongqing Medical University, The Key Laboratory of Biochemistry and Molecular Pharmacology, Chongqing 400016, China

**Keywords:** aluminum overload, hippocampal neuron, PGE_2_, PGE_2_ receptor

## Abstract

To observe the characteristic changes of PGE_2_-EP_s_ pathway and divergent functions of PGE_2_ receptor subtypes on neuronal injury. The primary cultured rat hippocampus neuron injury model was established via aluminum maltolate (100 μM). The aluminum-overload neurons were treated with the agonists of EP1 (17-phenyl trinor Prostaglandin E2 ethyl amide), EP2 (Butaprost), EP3 (Sulprostone) and EP4 (CAY10598) and antagonists of EP1 (SC-19220), EP2 (AH6809) and EP4 (L-161982) at different concentrations, respectively. The neuronal viability, lactate dehydrogenase leakage rate and PGE2 content were detected by MTT assay, lactate dehydrogenase assay kit and enzyme-linked immunosorbent assay, respectively. The mRNA and protein expressions of mPGES-1 and EPs were determined by RT-PCR and western blot, respectively. The pathomorphology was identified by hematoxylin-eosin staining. In the model group, neuronal viability significantly decreased, while lactate dehydrogenase leakage rate and PGE2 content increased. The mPGES-1, EP1, EP2 and EP4 mRNA expression, and the mPGES-1, EP1 and EP2 protein expression increased, while EP_3_ level decreased. EP3 agonist exerted protective function in neuronal viability and lactate dehydrogenase leakage rate, while EP1 agonist, EP2 and EP4 antagonist exerted an opposite effect. In conclusion, aluminum-overload caused an imbalance of PGE_2_-EP_1-4_ pathway and activation of EP receptor may provide a viable therapeutic target in neuronal injury.

## INTRODUCTION

Neurodegenerative diseases (NDDs), including Amyotrophic lateral sclerosis (ALS), Huntington's disease (HD), Parkinson's disease (PD), Alzheimer's disease (AD), Spinal muscularatrophy (SMA) and related neurological and psychiatric disorders, occur frequently in the elderly. Owing to the complexity of the pathogenesis, pathogenic sites and different course of NDDs, effective preventive and treatment strategy hadn't been found. AD is an aging-associated, chronic, progressive disease characterized by three major neuropathological features: extracellular amyloid beta (Aβ) deposition, intracellular neurofibrillary tangles (NFTs) and selective neuronal loss [1]. As widely recognized, chronic inflammation is a key contributor to the pathology and progress of AD [2]. Epidemiological studies indicated that the application of non-steroidal anti-inflammatory drugs (NSAIDs) could reduce the morbidity and progression of AD [3].

Our previous studies showed that meloxicam significantly improved behavioral and biochemical changes in aluminum overloaded mice, which indicated that selective cyclooxygenase-2 (COX-2) inhibitors have potential values in chronic neuronal damage-related diseases [4]. AD and cognitive deficit could only be prevented by long-term used of selective COX-2 inhibitors, but not cured [5, 6], meanwhile the risk of complications, such as gastrointestinal ulcers, bleeding, cardiovascular and cerebrovascular diseases also increased [7]. To avoid such side effects, some scholars suggested that the downstream intervention of COX-2 (PG synthetase-PGs-PGs receptor signaling pathway) may be superior to upstream interference of COX-2 in the treatment of acute or chronic brain injury [8].

PGE2 is an important pro-inflammatory factor in the downstream of COX-2, which is one of the most widely distributed PGs. Prostaglandin E synthase (PGES) catalyzes prostaglandin H2 to generate PGE2, which has three subtypes, microsomal PGES-1(mPGES-1), microsomal PGES-2(mPGES-2) and cytosolic PGES(cPGES). Inducible mPGES-1 takes effect by coupling with COX-2 in pathological processes. PGE2 not only protects against neuronal lesion, but also involved in both ischemic and excitotoxicity brain injury. The reason may be related to diverse receptor subtypes exist in the PGE2 EP receptor family and distribution, and depend on the primary stimulus belongs to inflammation or excitotoxicity, such as PGE2 is a protective prostaglandin in the inflammation injury model [9, 10].

Aluminum (Al) is a neurotoxin in central nervous systerm(CNS), both by systemic administration and direct intracerebral injection [11]. As one of the most distributed metal in the crust, aluminum is widely applied in daily life. Diet is a major way of human exposure to aluminum, meanwhile antiperspirants, antacids and vaccines are also important ingestion routes [12]. Aluminum is mainly deposited in the spleen, liver, kidney, heart as well as in various brain regions, including hippocampus and cortex [13]. Studies show that aluminum accumulates abnormally in the senile plaques and neurofibrillary tangles of AD patients’ cortex [14]. Prolonged exposure to high-dose aluminum could produce severe CNS toxicity, which manifested as behavioral and cognitive dysfunction, neuronal damage, even neuron degeneration, but the mechanism is still unclear.

In present study, we established the injury model of hippocampus neuron via aluminum overload to observe the characteristic changes of mPGEs-1-PGE2-EPs signaling pathway. Additionally, seven kinds of EP agonists and antagonists were used to investigate the function of EP1-4 receptors and to explore the potential mechanisms related to the neuronal injury induced by aluminum overload. The results of our present study will provide a reliable experimental and theoretical basis for the development of effective NDDs drugs.

## RESULTS

### Primary neuron culture and identification

**Figure 1:**
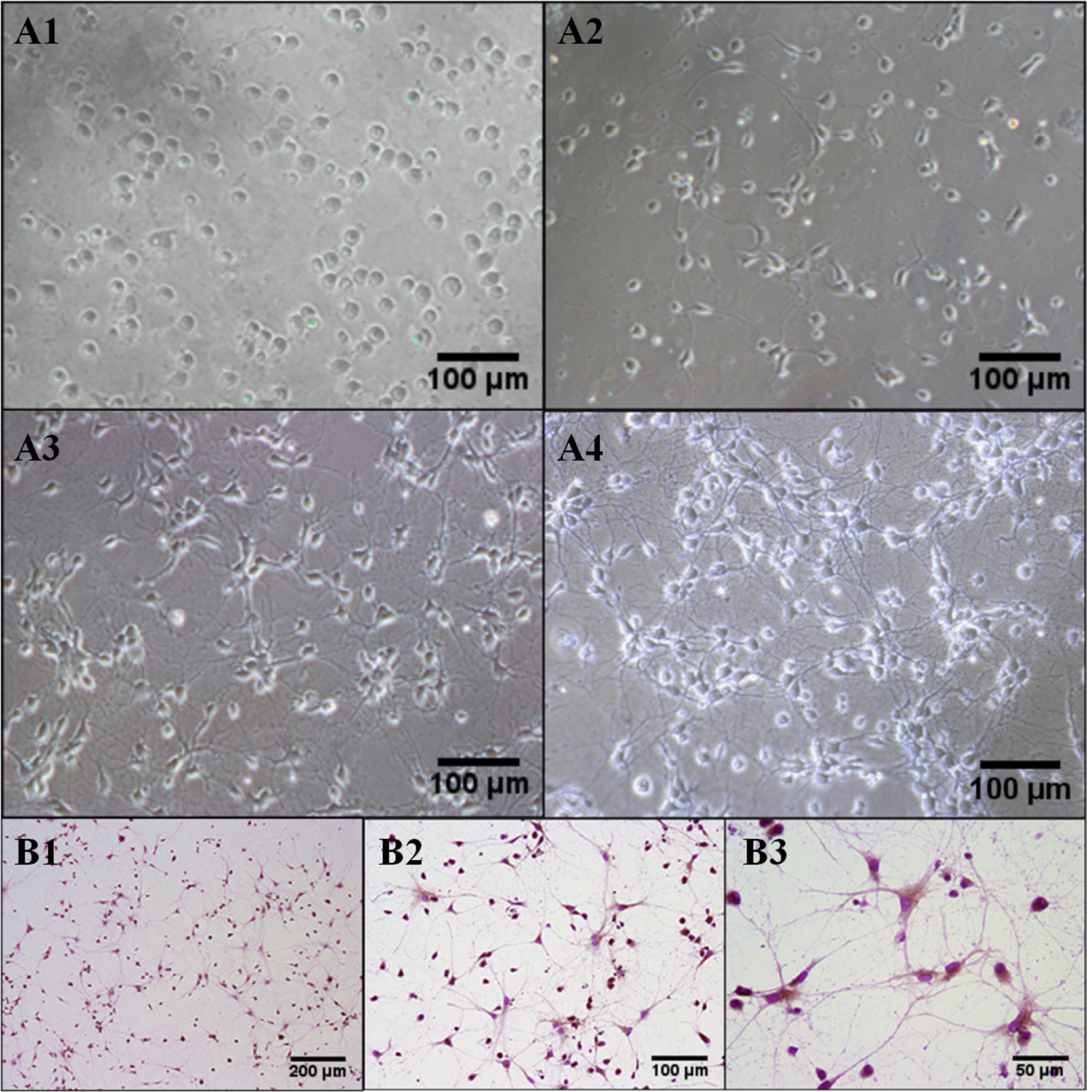
The morphology of primary cultured rat hippocampus neurons (**A1, A2, A3, A4**) After cultured for 1 d, 3 d, 5 d, 7 d, representative photographs were taken under inverted microscope and chosen to show the morphology of primary cultured rat hippocampus neurons. Cultured 7 days later, adjacent neurons had developed to mutual cross-linked cell. Sections were pictured at 200× power. (**B1, B2, B3**) NSE staining showed that more than 95% of positive neurons existed. Sections were pictured at 100×, 200 × and 400 ×, respectively.

Observed under inverted microscope, the neurons adhered firmly and a small amount of short synapses after inoculated for 1 day. After 3 days, neuronal synapses lengthened, soma enlarged and cell diopter increased. On the 5th day, the shapes of cell bodies were round or triangle which further swelled with halo, strong three- dimensional sense and synaptic interwoven into a sparse network. On the 7th day, soma became larger with halo, strong stereoscopic, faintly visible nucleolus profile and synaptic interwoven into a dense network with coarse dendrites and branches (Figure 1A). Neuron specific enolase (NSE) staning was used to detect the purity of the primary cultured rat hippocampus neuron after cultured for 7 days. The cytoplasm and axons of the positive neurons were stained to be yellowish brown. After nuclei were stained to be blue after counterstained with hematoxylin, nuclei were blue-purple. Six fields were selected randomly, each about 100 cells to calculate the number and percentage of NSE positive cells. The result showed that NSE positive cells are more than 95% and the primary cultured neuron can be used in the next experiments (Figure 1B).

### Inhibitory effects of Al(mal)_3_ on primary cultured hippocampal neuron

**Figure 2:**
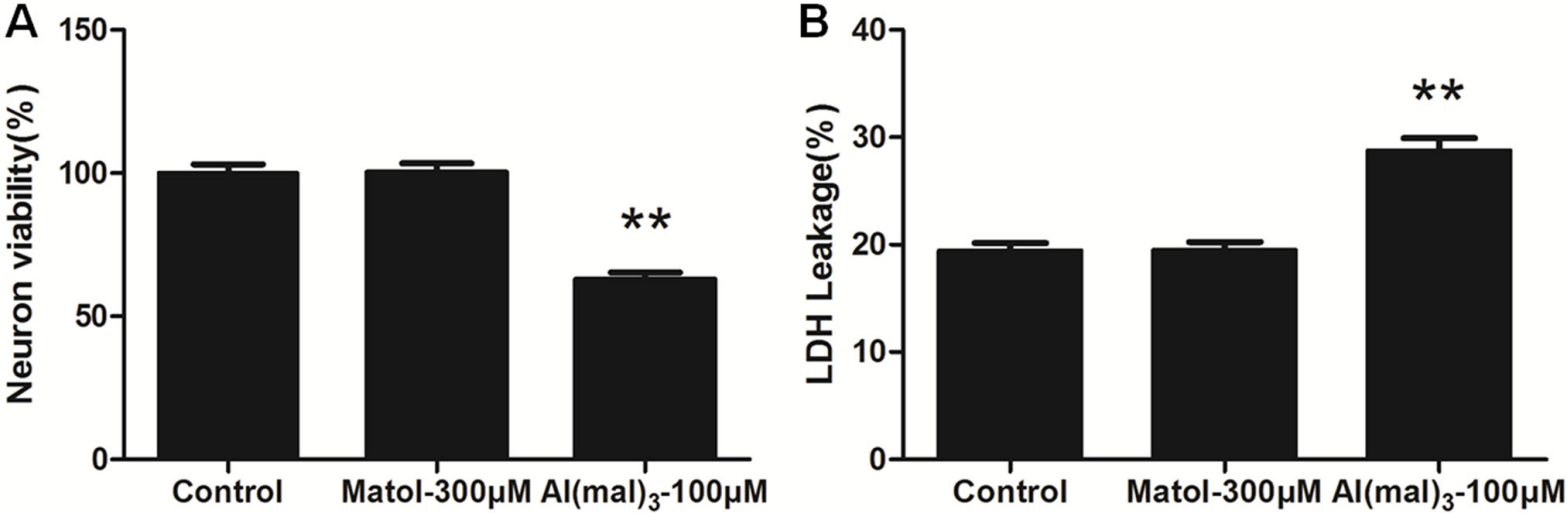
MTT assay and LDH leakage rate of primary cultured hippocampus neuron treated with Al(mal)_3_ and maltol (**A**) There were no significant differences in cell viability between the control group and 300 μM matol-treated group after 24 h of treatment. Whereas neuron viability showed a significant decreasing trend in the model group which treated with 100 μM Al(mal)_3_. (**B**) There were no significant differences in LDH leakage rate between the control group and 300 μM matol-treated group after 24 hrs of treatement. By contrast, LDH leakage showed a significant decreasing trend in the model group. Values were mean ± SD of ten individual experiments (*n* = 10, ^**^P < 0.01 vs. control group, one-way ANOVA with Dunnett's multiple comparisons).

Compared with the control group, MTT assay showed the neuron viability of 100μM Al(mal)_3_-treated group significantly decreased with the survival rate of 62.77%. However, the neuron survival rate was 100.2% at the concentration of 300 μM of maltol, therefore there was no significant difference between the solvent control group and the control group (Figure 2A). The result indicated that the solvent control group(300 μM maltol) has no significant difference in LDH leakage rates compared with the control group. LDH leakage rates increased significantly in 100 μM Al(mal)_3_ -treated group (Figure 2B).

### PGE2 levels detection by Enzyme-Linked Immunosorbent Assay (ELISA) in primary cultured hippocampal neuron

**Figure 3:**
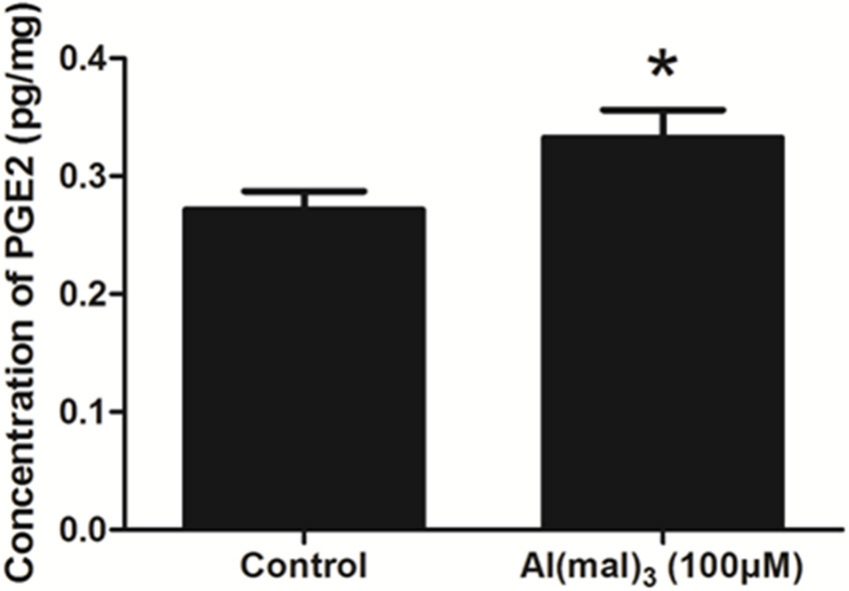
The content of PGE2 in each group detected by ELISA The content of PGE2 increased significantly in the Al(mal)_3_-treated group. Values were mean ± SD (*n* = 3, ^*^ < 0.05 vs. control group, Student's *t* test).

The concentration of PGE2 in control group and Al(mal)_3_-treated group was about 0.272 and 0.333 pg/mg, respectively. Compared with the control group, the content of PGE2 increased significantly in model group (*P* < 0.05) (Figure 3).

### Expressions of mPGES-1, EP1-4 mRNAs and proteins in primary cultured rat hippocampal neuron

**Figure 4:**
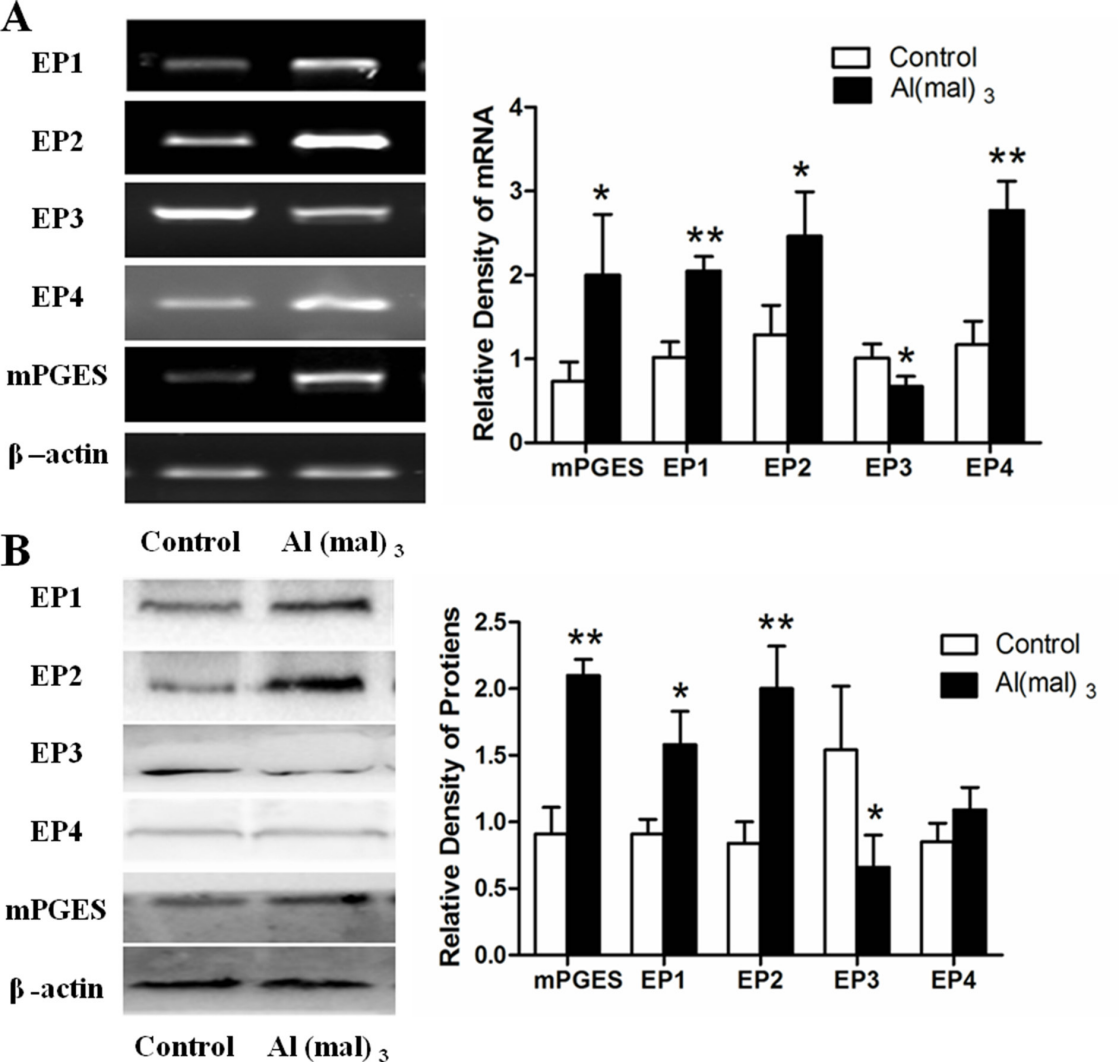
mPGES-1 and EP_1-4_ mRNAs and proteins expression in primary cultured hippocampus neuron (**A**) The expressions of mPGES and EPs mRNA were measured by RT-PCR. The relative mRNA level of mPGES and EPs were standardized to endogenous β-actin mRNA for each sample. Aluminum administration caused the significant increase of mPGES, EP_1_, EP_2_ AND EP_4_ levels and decrease of EP_3_ level compared with the control group. (**B**) The expressions of mPGES and EPs proteins were measured by WB. The relative protein levels of mPGES and EPs were standardized to endogenous β-actin protein for each sample. Al administration caused the significant increase of mPGES, EP_1_ and EP_2_ levels and decrease of EP3 level compared with the control group. Values were mean ± SD (*n* = 3, ^*^P < 0.05, ^**^P < 0.01 vs. control group, one-way ANOVA with Dunnett's multiple comparisons).

Compared with control group, the expressions of EP1 and EP4 mRNA incresed 100% (*P* < 0.01), and that of mPGES-1 and EP2 mRNA increased significantly in the model group which was treated with Al(mal)_3_ (*P* < 0.05). However, the expression of EP3 mRNA decreased more than 30% in the model group (*P* < 0.05) (Figure 4A). The result of WB showed that the expressions of mPGES-1 and EP2 proteins more than doubled in the Al(mal)_3_ treated group when compared with control group (*P* < 0.01), and that of EP1 proteins increased to 170% (*P* < 0.05), while the expression of EP4 proteins had no significant difference. By contrast, the expression of EP3 proteins decreased 50% in the model group (*P* < 0.05) (Figure 4B).

### MTT assay of aluminum-overloaded primary cultured hippocampal neuron treated by different concentrations of EP_1-4_ agonist or antagonist

**Figure 5:**
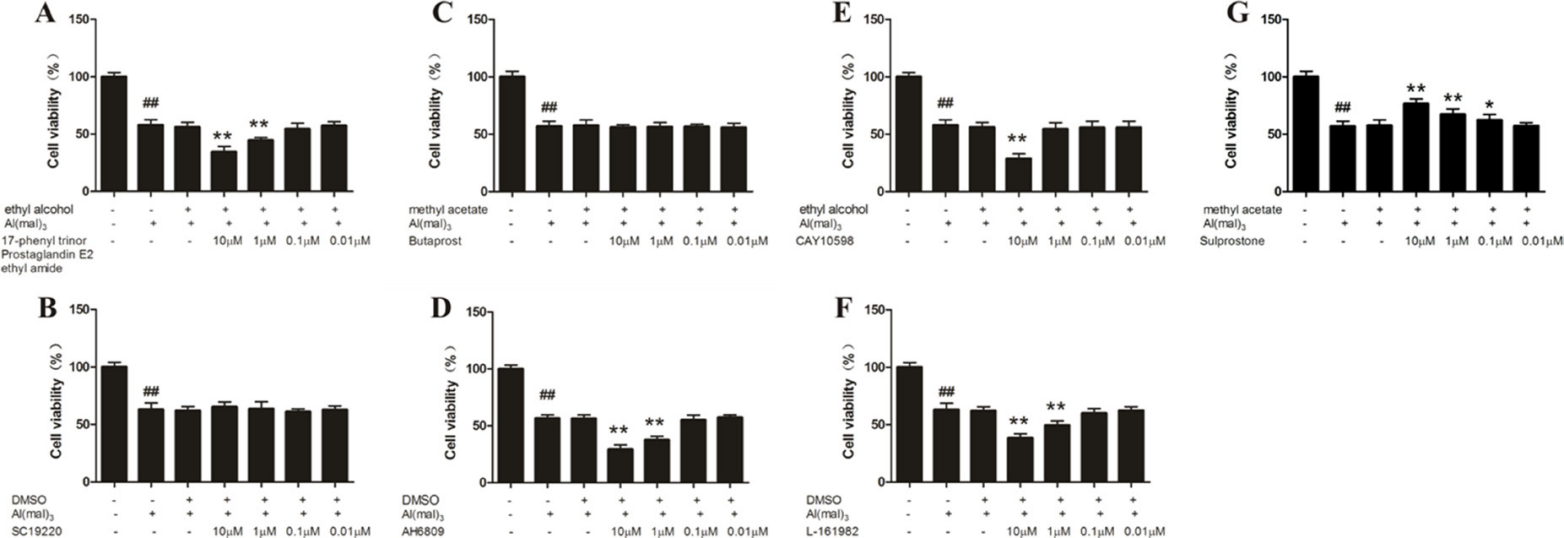
MTT assay of aluminum overload primary cultured hippocampus neuron treated by different concentrations of EP_1-4_ agonist or antagonist (**G**) Sulprostone increased neuron viability in a concentration dependent manner in Al^3+^-treated group, whereas (**A**) 17-phenyl trinor Prostaglandin E2 ethyl amide (**D**) AH6809 (**E**) CAY10598 and (**F**) L-161982 decreased neuron viability in Al^3+^-treated group. (**B**) SC-19220 and (**C**) Butaprost had no signifcant effect on the primary hippocampal neuron of rats treated with Al(mal)3. Values were mean ± SD of six individual experiments (*n* = 6, ^##^*P* < 0.01 compared with control group, ^*^P < 0.05 and ^**^P < 0.01 compared with Al^3+^-treated group, respectively, one-way ANOVA with Dunnett's multiple comparisons).

About the concentrations of the agonists and antagonists of EP, for the observation of neuronal viability, four concentrations (10^−5^, 10^−6^, 10^−7^, 10^−8^ M) of each agonists and antagonists of EP were used. The results of MTT assay showed that the neuron viability in the model group decreased significantly compared with the control group (*P* < 0.01). Compared with the model group, the viability of neuron treated with EP3 agonist (Sulprostone) at a concentration of 10 μM, 1 μM and 0.1 μM increased significantly (*p* < 0.01), whereas the administration of EP1agonist (17-phenyl trinor Prostaglandin E2 ethyl amide), EP2 antagonist (AH6809) and EP4 antagonist (L-161982) at a concentration of 10 μM and 1 μM and EP4 agonist (CAY10598) at a concentration of 10 μM decreased the neuron viability significantly (*P* < 0.01). The other concentrations of EP1 antagonist (SC-19220), EP2 agonist (Butaprost) and EP4 agonist (CAY10598) had no significant effect on the primary hippocampal neuron of rats treated with Al(mal)_3_. There was no considerable difference between the model group and the solvent control group (Figure 5).

### LDH leakage of aluminum overload primary cultured hippocampal neuron treated by different concentrations of EP_1-4_ agonist or antagonist

**Figure 6:**
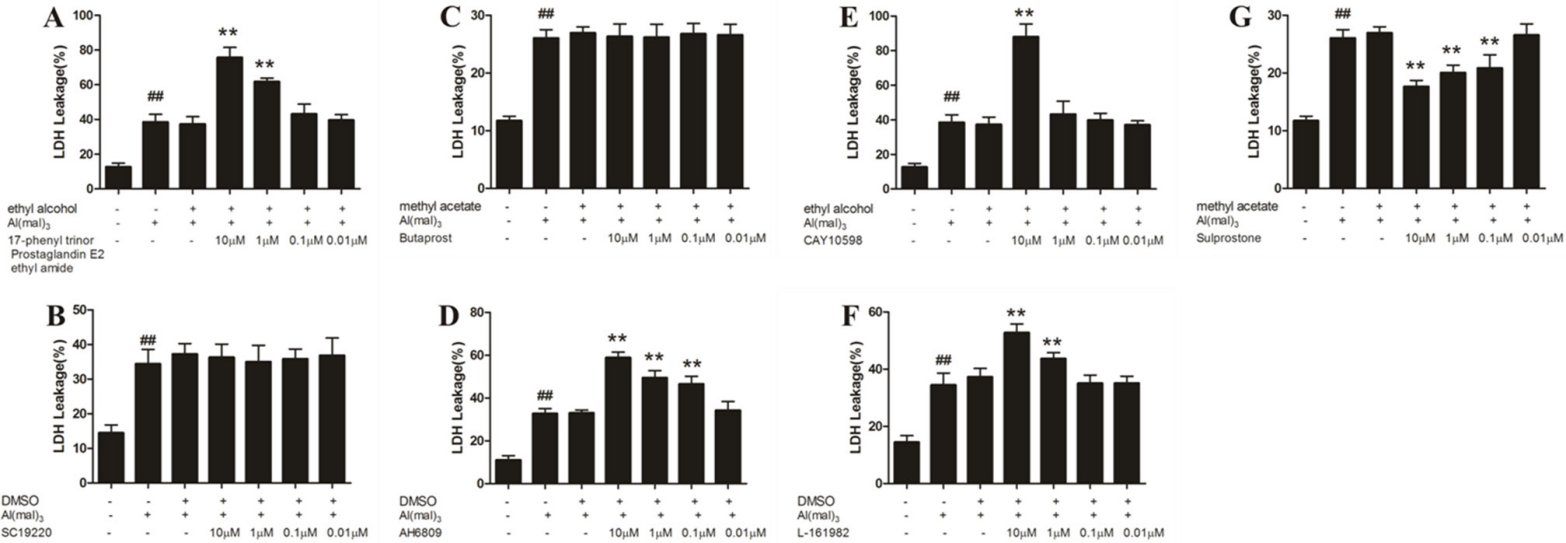
LDH leakage of aluminum overload primary cultured hippocampus neuron treated by different concentrations of EP_1-4_ agonist or antagonist (**G**) Sulprostone significantly decreased the LDH leakage rate in Al^3+^-treated groups, whereas (**A**) 17-phenyl trinor Prostaglandin E2 ethyl amide, (**D**) AH6809, (**E**) CAY10598 and (**F**) L-161982 decreased the LDH leakage rate significantly in Al^3+^-treated group. (**B**) SC-19220 and (**C**) Butaprost had no signifcant effect on the primary hippocampal neuron of rats treated with Al(mal)3. Values were mean ± SD of six individual experiments (*n* = 6, ^##^*P* < 0.01 compared with control group, ^*^P < 0.05 and ^**^P < 0.01 compared with Al^3+^-treated group, respectively, one-way ANOVA with Dunnett's multiple comparisons).

Compared with the control group, the LDH leakage rate increased significantly in the model group (*P* < 0.01). Compared with the model group, the LDH leakage rate of EP3 agonist (Sulprostone) at a concentration of 10 μM,1 μM and 0.1 μM decreased significantly (*P* < 0.01), whereas the administration of EP2 antagonist (AH6809) at a concentration of 10 μM,1 μM and 1 μM, EP1agonist (17-phenyl trinor Prostaglandin E2 ethyl amide)and EP4 antagonist (L-161982) at a concentration of 10 μM and 1 μM, and EP4 agonist (CAY10598) at a concentration of 10 μM increased the LDH leakage rate significantly(*P* < 0.01). The other concentrations of EP1 antagonist (SC-19220), EP2 agonist (Butaprost) and EP4 agonist (CAY10598) had no significant effect on the primary hippocampal neurons of rats treated with Al(mal)_3_. There was no considerable difference between the model group and the solvent control group (Figure 6).

### The morphological changes of aluminum overload primary cultured hippocampal neuron treated by different concentrations of EP_1-4_ agonist or antagonist

**Figure 7:**
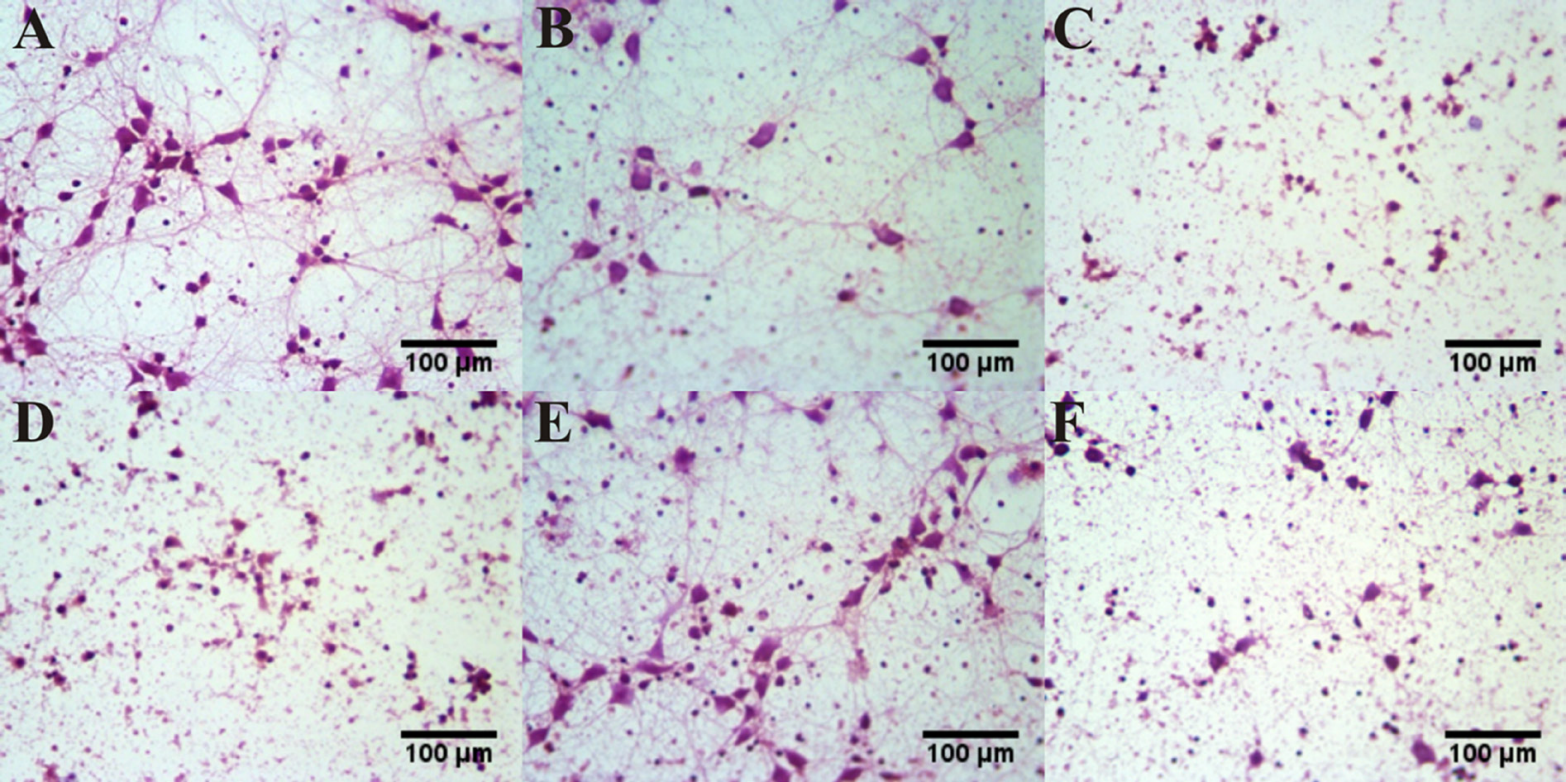
The morphological changes of aluminum overload primary cultured hippocampus neuron treated by different concentrations of EP_1-4_ agonist or antagonist The photograph showed the number and structure of neurons in (**A**) control group (maltol 300 μM) and (**B**) aluminum overload group (Al(mal)_3_100 μM). (**C**) Al(mal)_3_100 μM + 10 μM 17-phenyl trinor Prostaglandin E2 ethyl amide-treated group, (**D**) Al(mal)_3_100 μM + 10 μM AH6809-treated group and (**F**) Al(mal)_3_100 μM + 10 μM L-161982–treated group decreased the number and structure of neurons in the model group, whereas (**E**) Al(mal)_3_100 μM + 10 μM Sulprostone-treated group increased the number and structure of neurons in the Al(mal)_3_-treated group. Sections were pictured at 400×.

Compared with the control group, the neuronal synapses were degenerated and the number was decreased substantially with abundant scatterred cell debris in the aluminum load model group. Compared with the aluminum load model group, the number and synapses of hippocampal neurons were further decreased and exhibited karyopyknosis and disruption in the EP1 agonist, EP2 antagonist and EP4 antagonist treated group. By contrast, treated with EP3 agonist, the somata were complete and clear, the number and protrusions increased and cell decris reduced when compared with the model group. (Figure 7).

## DISCUSSION

Aluminum, an accepted metallic neurotoxin, is extensively used in everyday life. Foods, beverages (including water) and aluminum additives currently are considered as the main form of aluminum orally ingested by the populations [15]. It has been reported that aluminum accumulation is related to neurodegenerative diseases, such as Alzheimer's disease, Parkinson's disease and so on [16–18]. Bhattacharjee *et al*. found that the aluminum concentration in AD patients’ cerebral arteries and hippocampus is higher than that of age-matched non-dementia patients [19]. Aluminum induced selective neuronal death was due to the abnormal accumulation in the senile plaques and neurofibrillary tangles of AD patients’ cortex [14, 20, 21]. Walton presented the evidence that chronic low-level aluminum ingestion contribute to cognitive deterioration and associated pathology changes in a translation rat model [22]. *In vitro* study, aluminum initiated inflammatory in a human glioblastoma cell line, which can be chalked up to the activation of the immune-responsive transcription factor NF-κB and elevation of TNF-α in a time-dependent manner [23]. Johnson *et al*. found that Al(malt)_3_ can cause apoptosis in a murine neuroblastoma cell line (Neuro-2a cells) accompanied by a marked increase of p53 expression and inhibition of Bcl2 expression [24]. Aluminum accumulation in Neuro 2A cells could increase Fe uptake and NFT protein expression and inhibit the cell growth [25]. We previously found that chronic aluminum exposure via intragastric administration caused significant increase of metal ion (Al, Fe, Mn, Cu and Zn) and related oxidative stress levels in rat hippocampus, and induced learning and memory function disorders [26].

Aluminum maltolate (Al(mal)_3_) is superior to other salt forms of aluminum used *in vitro* mechanistic studies because it doesn't form insoluble aluminum hydroxide precipitates at physiological pH [24, 27]. At present, animal model is regularly used for studying on the neurotoxicity of aluminum, so the injury of primary cultured hippocampal neurons treated with Al(mal)_3_ *in vitro* was used to simulate neurodegenerative diseases in our study. In the present study, the viability of neurons measured by MTT assay were decreased significantly, while the LDH leakage rates were significantly increased, in contrast to the control group. These results indicated that the injury model was established successfully.

The type and pathogenesis of neurodegenerative diseases are complicated, such as AD currently existing amyloid hypothesis, oxidative stress hypothesis, brain inflammation hypothesis and tau protein hypothesis. Recent studies found that aluminum administration can elevate the activity of superoxide dismutase (SOD) in all brain regions, providing unequivocal evidence that oxidative stress is related to aluminum neurotoxicity [28]. Recent studies show that inflammation plays a dual role in the process of neurodegeneration disease. On the one hand, the inflammatory response can aggravate the process of neurondegeneration [29], but on the other hand, inflammation has a neuroprotective effect [30]. In APPswe/PS1 transgenic mouse model, microglia was activated by inflammatory cytokines such as IL-1β, TNF-α and IL-6 to phagocytose β-amyloid for protecting neurons [30]. Our previously study confirms that the disorder of cyclooxygenase-2 downstream (PGS/PGs/PG receptors) signaling pathway in rat hippocampus caused by chronic aluminum overload, illustrating that inflammatory is involved in aluminum neurotoxicity [31]. We previously found that the chronic aluminum gluconate administration induced cerebral injury and neurodegeneration in rats, while symptom was ameliorated by meloxicam, a COX-2 inhibitor [32]. These findings reveal that inflammatory mediators COX-2 is a vital factor in neuron injury and neurodegeneration, and the inhibitors of COX-2 may prevent the chronic aluminum overload induced brain damage.

Prostaglandin E2 (PGE2) is one of the most important members of the PGs family, and distributed widely in the body [33]. PGES is a vital rate-limiting enzymes for the synthesis of PGE2. According to the cellular localization of PGES and GSH dependence, PGES can be divided into 3 subtypes, cPGES, mPGES-1 and mPGES-2 [34]. Inducible mPGES-1 could be preferentially coupled to COX-2, which promoted the response to various stimulations and PGE2 generation [34]. According to research, the levels of cerebrospinal fluid PGE2 increased in mild memory impairment patients, but decreased in those with advanced Alzheimer's disease [35]. PGE2 has been described to exert either neurotoxicity or neuroprotection in the cerebral injury due to diverse receptor subtypes and different disease models [33, 36]. The results of the present study showed that the expressions of mPGES-1 mRNA and protein in the aluminum overload group were significantly increased compared with control group, suggested that mPGES-1 may be participated in neuronal degeneration. Meanwhile, our experimental results showed that the PGE2 level increased significantly in the primary neurons treated with Al(mal)_3_. The results indicated that the increase of mPGES-1 expression results in the rise of PGE2 level and mPGES-1, PGE2 may exert neurotoxicity effect. We propose that downstream intervention of PG receptors may be a great therapeutic target of AD.

Four distinct EP receptors with different ligand affinity (EP3 > EP4>EP2 > EP1) and expression profile, coupling to opposing second messengers which contribute to opposing actions in tissues and cells [36, 37]. EP1 is mainly expressed in the hypothalamus and thalamus, cortex, hippocampus, cerebellar neurons and microglia, EP2 is widely expressed in the forebrain, brainstem, thalamus, hypothalamus and spinal cord, EP3 is widely expressed in the subcortical structures, especially in thalamus and EP4 was expressed mainly in the forebrain and thalamus neurons, astroglia and a small quantity of endotheliocytes [36, 38–43]. PGE2 binds to EP1 and EP2 with much lower affinity than EP3 and EP4. PGE2 exerts its downstream effects by activating EP receptors (EP1-4) that have diverse effects on kinase and second messenger including IP_3_, cAMP and Ca^2+^ [36, 37, 44]. The rat EP1, EP2, EP3 and EP4 receptors encode proteins of 405, 357, 390 and 488 amino acids, respectively. Because of the diverse C-terminal sequence EP3 can be divided into EP3α, EP3β, and EP3γ [36, 44].

The structure of 17-phenyl trinor prostaglandin E2 ethyl amide is similar to that of 17-phenyl trinor prostaglandin E_2_, which can selectively excite EP1, EP2 and EP3 receptors, especiallyEP1 [45, 46]. EP1 activation was involved in the stress response, the promotion of the carcinogenic role of chemicals, inflammation, anaphylactic reaction, epilepsies and neurodegeneration [47–51]. SC-19220 is a kind of selective EP1 antagonist, which is related to the triggering of PGE2 induced pain, the growth and survival of neurons, and the inhibition of tumor metastasis [52]. In a rodent model of Parkinson, SC-19220 (1.5 μM) can prevent the loss of dopaminergic neurons in the substantia nigra induced by PGE2. Butaprost binds with about 10% the affinity of PGE2 to the recombinant murine EP2 receptor, and does not bind appreciably to any of the other murine EP receptors or DP, IP, FP or TP receptors. AH6809 is an EP2 receptor antagonist with high affinity. EP2 is widely distributed in the human body and participates in a variety of physiological and pathological processes, which is thought to be related to the transmission and plasticity of synapses in CNS and promotes the deposition of β -amyloid in the brain of AD transgenic mice [53, 54]. EP3 is mainly involved in pathophysiological processes, such as fever, anaphylaxis, tumor angiogenesis and chronic inflammation. Sulprostone is an EP3 selective agonist (KI = 0.6), but also partially excites EP1 (KI = 21), CAY10598 specifically stimulates EP4 receptor (KI = 1.2 nm) with intensive affinity, L-161982 mainly antagonizes EP4 receptor and also has some weak antagonistic effects on other PGs receptors. Studies reported that the first generation of EP4 agonists can reduce inflammation response, aid in the healing of bones, protect the cardiovascular and cerebral cells, and reduce the renal dysfunction [55–58].

Studies showed that EP1 receptor mainly cause severe neurological and functional deficits in the nervous system diseases. Different from other EP receptors, EP1 coupled with Gαq and the concentration of IP3 and Ca^2^+ increased after the activation of EP1 [59]. Previous study shown that deletion of EP1 receptor and intervention of EP1 antagonist attenuate cerebral injury induced by NMDA and MCAO and the agonist exacerbates cerebral injury induced by NMDA. EP1 receptor deletion attenuates 6-OHDA-induced Parkinsonism by protecting the dopaminergic neurons. EP1 knockout can also protect the APP/PS1 mice neurons and reducing beta amyloid protein precipitation [60]. These researches indicate that EP1 receptor involves in the pathogenesis of neurodegenerative diseases (NDDs). We found that the increase of EP1 mRNA and protein expression in aluminum overload group were significant compared with control group. Whereas EP1 agonist (17-phenyl trinor Prostaglandin E2 ethyl amide) treatment significantly decreased neurons’ viability and increased LDH leakage rate contrast to aluminum overload group, while EP1 antagonist (SC-19220) treatment had no significant effect on the viability and LDH leakage rat. Our results indicated that the neurotoxicity role of aluminum was correlated to the PGE2-EP1 pathway.

EP2 is a kind of G_S_-protein coupled receptors, which occupies an important position in neuroinflammation and neurodegenerative diseases [61, 62]. The study showed that EP2 receptor was highly expressed in LPS induced spinal cord neuron inflammation model rat and in the brain of APPSwe /PS1 mice. Moreover, deletion of EP2 receptor also decreases expression of proinflammatory factors such as COX-2, iNOS and NADPH oxidase subunits in hippocampus. EP2 show different effects in distinct disease models. In APPSwe/PS mouse model, EP2 knockout can significantly reduce the expression of inflammatory cytokines in mice brain [63], while EP2 agonist can also reduce oxidative stress and mortality in primary cultured neurons treated with β - amyloid protein [64]. McCullough reported that activation of EP2 receptor can protect against toxicity in ischemia, hypoxia and excitotoxic models in a cAMP dependent manner, while genetic deletion of EP2 receptor can exacerbate infarction of cerebral cortex and subcortical structures [65]. Our previous study demonstrated that PGES-PGE2-EP2 signaling pathway disorders might have a vital role in cerebral injury and neuronal degeneration rats caused by chronic aluminum overload, while the mechanism of which is still unknown [66]. In the present study, EP2 was highly expressed in aluminum overload primary hippocampal neurons, whereas treatment with EP2 antagonist (AH6809) aggravated the aluminum-overload damage.

As with the EP2 receptor, EP4 is a Gs-coupled receptor and the content of cAMP can be up-regulated after the stimulation. EP4 can also activate phosphatidylinositol 3-kinase (PI3K) signaling via coupling with the Gi protein [67]. Different from EP2, most studies showed that EP4 provided protection effects to the nervous system. For example, activation of EP4 receptor could protect against neuroinflamation via down -regulating TNF, COX-2 and mPGES-1 content in the spinal neurons of mPGES-1- deficient mice induced by LPS [68]. Liang reported that EP4 agonist reduces infarct volume and ameliorates long term behavioral deficits after ischemia, whereas genetic inactivation of EP4 worsened stroke outcome [69]. Contrary to the studies, our previous study demonstrated that PGES-PGE2-EP4 signaling pathway disorders might related to cerebral injury and neuronal degeneration rats caused by chronic aluminum overload [66]. In present study, the expression EP4 mRNA in aluminum overload group significantly increased compared with control group. Treatment with EP4 antagonist (L-161982) significantly worsen the neuronal injury induced by aluminum overload. While, treatment with EP2 agonist and EP4 agonist had no significant effect on the viability and LDH leakage rates. Therefore, the significance and mechanism of both EP2 and EP4 in aluminum-overload induced neuronal injury need to be further investigated.

In mouse, alternative splicing of the C-terminal tail creates three EP3 splice variants, α, β, and γ. The major signaling pathway of the EP3 receptor is inhibition of cAMP via coupling to Gi, activation of IP3/Ca^+^ pathway by coupled with Gq under specific circumstances, and it also coupled with G12 to activate Rho-kinas [70]. Due to the complexity mentioned above, the activation of different EP3 splice variants could lead to opposite functions. Numerous studies stated that EP3 receptor is considered to exert neuroprotective effects [71]. Selective EP3 agonist sulprostone can decrease the necrosis of neurons in chronic glutamate toxicity model. The protective mechanism may be related to coupling with Gi protein [39]. Wu *et al*. found that stimulation of EP3 receptor by sulprostone resulted in a significant rescue of CA1 neurons in the hippocampal slices treated with NMDA [9]. Previous study indicate that the activation of PGE2-EP3 signaling contribute to neuroprotective effect on neurodegeneration of APP/PS1 mice [66, 72]. However, some investigator state that EP3 aggravates neurotoxicity. Ahmad found that a selective EP3 agonist, ONO-AE-248, significantly increases infarct size in the MCAO model. Pretreatment with ONO-AE-248 exacerbates the acute excitotoxicity of NMDA, while deletion of EP3 ameliorates the neurotoxicity, suggesting that PGE2 can contribute to the neurotoxicity of COX via EP3 [73]. Different functions among these EP3 splice variants have been reported, including coupling to different signal transduction pathways, distinct sensitivities to agonist-induced desensitization, different in agonist-independent constitutive activity, diverse intracellular trafficking patterns, and varied agonist-induced internalization patterns [70, 74, 75]. Furthermore, ONO-AE-248 is highly selective toward EP3 receptor, whereas Sulprostone is listed as a comparatively weak agonist of EP1 receptor in addition to the high affinity of EP3 receptor and also has the advantage of being relatively resistant to metabolically degraded. In our experiment, we found that compared with the control group, EP3 expression in aluminum overload group significantly decreased. EP3 agonist (Sulprostone) significantly reserved the decrease of viability and the increase of LDH leakage rate. These results in the present study suggested that EP3 may mediate the neuroprotection roles of PGE2 in AD. However, the EP3 antagonist was not used in the present study. Therefore, further studies are needed to target at EP3 receptor and splice variants.

Based on the related researches [76, 77], Al(mal)_3_(100 μM)was chosen to establish aluminum overload model in primary hippocampal neurons. The expression of mPGES1, EP1, EP2 and EP4 increased while EP3 decreased in the model induced by aluminum. The results suggest that mPGES-1-PGE2-EP1-4 receptor signaling pathway disorder in aluminum overloaded primary hippocampal neurons, which may be an important mechanism leading to aluminum damage to neurons. Our study also showed that EP receptors intervened, including activation of EP1 receptor as well as antagonism of EP2 and EP4 receptors could increase the susceptibility of hippocampal neurons to aluminum toxicity, whereas the neuron susceptibility to aluminum toxicity significantly decreased by the activation of EP3 receptor. These results suggest that EP1 may mediate the neurontoxicity of aluminum overload in primary hippocampal neurons, while EP2, EP3 and EP4 may mediate a protective mechanism to aluminum toxicity. EPs may be a new candidate target for the intervention of neurodegenerative diseases and the search for protective drugs. However, considering the complexity of COX-2 downstream pathway, the specific regulation mechanism of EP in CNS is worth our further study.

## MATERIALS AND METHODS

### Animals

All the experimental procedures were approved by the Animal Laboratory Administrative Center and the Institutional Ethics Committee of Chongqing Medical University (License number: SYXK YU 2012-0001) and also in accordance with the National Institutes of Health guidelines. Rats were housed in the barrier housing facility, and it has in keeping with national standard “Laboratory Animal- Requirements of Environment and Housing Facilities”. The care of laboratory animal and the animal experimental operation have conforming to “Chongqing Administration Rule of Laboratory Animal”.

### Chemicals

AlCl_3_·6H_2_O (*Sinopharm Chemical Reagent Co., Ltd., China*) and maltol (*Aladdin*, USA) were of analytical grade. Maltol solution(60 mM) was prepared by adding 0.3784g maltol into 50 ml of autoclaved PBS and 0.1207g AlCl_3_·6H_2_O was added into 25 ml of autoclaved PBS as AlCl_3_ solution(20 mM). Aluminum-maltolate solution (10 mM) was prepared by adding 25ml maltol solution and 25 ml AlCl_3_ solution. After filtered through 0.22 mm millipore filter, 60 mM maltol solution and 10 mM aluminum-maltolate solution was stored at 4°C until used [24, 78]. SC19220, AH6809 and L-161982 (*Cayman, USA*) were dissolved in the DMSO (*Sigma*, *USA*) to be 10 mM reserve liquid. 17-phenyl trinor Prostaglandin E2 ethyl amide and CAY10598 (*Cayman, USA*) were dissolved in ethyl alcohol to be 10 mM reserve liquid. Butaprost and Sulprostone (*Cayman, USA*) were dissolved in methyl acetate to be 10 mM reserve liquid [24].

### Rat primary hippocampal neuron culture

Primary hippocampal neurons were prepared from E18 Sprague-Dawley (SD) rat embryos and were immediately soaked with 75% ethanol. The hippocampus was dissected from the brain of each rat on ice and stripped the meninges and blood vessels carefully with sterile phosphate-buffered saline (PBS) washing. The tissues were minced and digested with 0.125% trypsin at 37°C for 20 min; digestion was stopped by adding DMEM/F12 medium with 10% fetal bovine serum (FBS) (*Gibco, USA*). The neurons were centrifuged and suspended to a density of 1 × 10^6^/L in DMEM/F12 (*HyClone, USA*) with 10% FBS. The different volumes of neuronal suspensions were inoculated in culture flasks and coated with L-poly lysine (*Sigma, USA*) and cultured in a humidified 5% CO2 atmosphere at 37°C. When the neurons adhered, the medium was changed to neurobasal medium (*Gibco, USA*). Half of the medium was changed every 3 days. After cultured for 48 h, cytarabine (4 mg/ml) was injected to inhibit the growth of glial cells. Cultured neurons were used for *in vitro* studies on the 7th day.

### Neuron-specific enolase identification

Hippocampal neurons were inoculated in the 6-well plates with circular coverslips until D7 for identification. Neurons were washed three times with PBS, 2 min per time and fixed with 4% paraformaldehyde for 30 min at 4°C. The activity of endogenous peroxidase in the neurons was inhibited with 3% H_2_O_2_ for 15 min. Non-specific binding of neurons was blocked with Goat serum (10%) for 20 min at room temperature after three times washed with PBS. Then, neurons were incubated with specific primary antibody of NSE (neuron-specific enolase, 1:50, *Bostor, China*) overnight at 4°C. Afterward, the neurons were incubated with the second antibody (biotin-labeled goat anti-rabbit) at 37°C for 30 min and with horseradish peroxidase-labeled avidin for 30 min at 37°C. DAB (*ZSGB, China*) was used for coloration, and the samples were counterstained with hematoxylin, transparent in xylene and sealed with neutral gum.

### Models establish

On the seventh day, the neurons were plated in 96-well culture plates at a density of 1 × 10^5^ neurons/ml. Hippocampal neurons were divided into the blank control group(PBS), control group(maltol 300 uM) and aluminum overloaded group(Al(mal)_3_ 100 uM). After cultured for 24 h at 37°C and 5% CO2, the relevant indicators can be detected.

### EP receptors intervention

The neurons were plated in 96-well culture plates at a density of 1 × 10^5^ neurons/ml. The neurons were divided into the control group (maltol 300 μM), the aluminum overloaded group (Al(mal)_3_ 100 μM), the solvent control group (solvent+ Al(mal)_3_ 100 μM) and the intervention groups were established base on aluminum overloaded. Seven intervention groups including EP1 agonist (17-phenyl trinor Prostaglandin E2 ethyl amide), EP1 antagonist (SC-19220), EP2 agonist (Butaprost), EP2 antagonist (AH6809), EP3 agonist (Sulprostone), EP4 agonist(CAY10598) and EP4 antagonist (L-161982) were added up into the intervention group respectively with various concentrations (10^−5^, 10^−6^, 10^−7^, 10^−8^ M) for 24 hours at 37°C and 5% CO2. The solvent control of Butaprost and Sulprostone intervention groups was an equivalent of 0.35‰ methyl acetate and the solvent control of 17-phenyl trinor Prostaglandin E2 ethyl amide and CAY10598 intervention groups was supplied with equal amount of0.25‰ ethyl alcohol. However, the equal amount of 0.5‰ DMSO as the solvent control of SC-19220, AH6809 and L-161982 intervention groups.

### Cell viability assays

The primary cultured hippocampal neuronal viability was determined by 3-(4, 5-Dimethyl-thiazol-2-yl)-2, 5-diphenyl-tetrazolium bromide (MTT) (*Sigma, USA*) assay, the amount of formazan was proportional to the number of living cells. The cultured rat hippocampal neurons in the 96-well culture plate were cultured until D7 for Al(mal)_3_ or drug treatment. First, 20 μl of 5 mg/ml MTT was added to each well and away from light. After cultured for 4 h at 37°C and 5% CO2, the media was carefully removed and 150 μl DMSO was added into each well for dissolving the insoluble formazan. Then, the plate shook slowly on the horizontal shaking table free from light for 10 min at room temperature. Finally, optical density (OD) was detected at 490 nm by a microplate reader (*BioTek, USA*) [79].

### Lactate dehydrogenase (LDH) leakage rate measurement

The cultured rat hippocampal neurons in the 96-well culture plate were cultured until D7 for Al(mal)_3_ or drug treatment and cultured for 24 hours at 37°C and 5% CO2. Finally, the LDH test kit was used according to the manufacturer's instructions (*Beyotime, China*) [79].

### PGE2 level detection by Enzyme-Linked Immunosorbent Assay (ELISA)

The rat hippocampal neurons were inoculated in the culture flasks and divided into the control group and aluminum overloaded group until D7. After 24 h intervention, neurons were collected after trypsin digestion. Neurons were disrupted by ultrasonic with indomethacin solution and centrifuged for supernatants. Collected cell supernatants stored at −80°C until used with the methods. PGE2 concentration in the supernatant was detected by ELISA kits (*Cloud-Clone Corp, USA*), according to the manufacturer's specifications.

### Reverse Transcription Polymerase Chain Reaction (RT-PCR)

Total RNA was extracted from rat primary neurons according to RNAiso Plus reagent (*Takara, China*), cDNA templates was synthesized by PrimeScript^®^ 1st Strand cDNA Synthesis Kit (*Takara, China*) following the manufacturer's instructions, and then amplified by using the Premix PCR kit (*Cwbio, China*). PCR products were separated by 2% agarose gel electrophoresis. All the samples were normalized by the expression level of β-actin. The optical density (OD) values were measured with a Bio-Rad gel imaging analysis system (*Bio-Rad, USA*). According to GenBank mPGES-1, EP1, EP2, EP3, EP4 mRNA sequence of rats and design primers. Target genes and reference gene (β-actin) primers by the Sangon biotech design and synthesis, primer sequences are listed in Table 1.

**Table 1:** Primers for qPCR

Primer	Sequence (5′ to 3′)	Product Size
mPGES-1	Forward: 5′- GTGATGGAGAACAGCCAGGT -3′ Reverse: 5′- TGAGGACCACGAGGAAATGTA -3′	307 bp
β-actin	Forward: 5′-ACGGTCAGGTCATCACTATCG-3′ Reverse: 5′-GGCATAGAGGTCTTTACGGATG-3′	155 bp
EP_1_	Forward: 5′-CAAACCATTCTGGGCTCAAG-3′ Reverse: 5′-ACAGAGGTGGGACGTGAATC-3′	303 bp
EP_2_	Forward: 5′-AATGCGCTCAGTCCTCTGTT-3′ Reverse: 5′-GCCACCCGATGTGAACTTTA-3′	310 bp
EP_3_	Forward: 5′-GGTCGCCGCTATTGATAATG-3′ Reverse: 5′-TGAGGCCGAAAGAAGATACAA-3′	311 bp
EP_4_	Forward: 5′-CAGCCAAGTGTGGTGAAAGA-3′ Reverse: 5′-GGCAGGTATAGGAGGGTCTG-3′	302 bp

### Western blotting

After treatment, neurons from each group were collected and lysed by RIPA Lysis Buffer on ice, then centrifuged for supernatant at 12,000 × g for 10 min at 4°C and the protein concentrations were determined with a BCA protein assay kit (*Beyotime, China*). The remaining supernatant were mixed with loading buffer and boiled in 100°C for 10 minutes. Ultimately, samples were stored in –20°C for further analyses.

Equal amounts of protein (20 mg) was fractionated by sodium dodecyl sulfate polyacrylamide gel electrophoresis (SDS-PAGE) and then transferred to PVDF membranes (*Millipore, USA*). Non-specific binding was blocked with 5% skimmed milk in TBST buffer for 1 h at room temperature. Blots were probed with specific primary antibodies against m-PGEs, EP1, EP2, EP3, EP4 (1:200; *Cayman, USA*), and β-actin (1:1000; *Boston, China*) overnight at 4°C, after that they were washed for three times with TBST, 10 min per time. The membranes were incubated with HRP-conjugated secondary antibodies (1:3000) at room temperature for 1 h, after that they were washed for three times with TBST, 10 min per time. Finally, protein signals were visualized by ECL (*Bio-Rad, USA*) and quantification and statistical analyses of data were measured with Image Lab. All experiments were confirmed in triplicate.

### Observation of pathological morphology

Primary cultured rat hippocampal neurons were inoculated in the 12-well plates at a density of 7 × 10^5^ cells per well with circular coverslips until D7 for drug treatment. The neurons were divided into six groups, including the control group (maltol 300 μM), aluminum overload group (Al(mal)_3_ 100 μM) and the intervention group. 10^−5^M of 17-phenyl trinor Prostaglandin E2 ethyl amide, AH6809, Sulprostone and L-161982 were added into the intervention group, respectively. After 24 h of treatments as mentioned above, the culture medium was discarded and the neurons were rinsed 3 times with PBS, fixed with 4% paraformaldehyde (PFA) for 15min and then washed with PBS. The neurons were stained with Hematoxylin-Eosin. Morphological changes of the neurons were observed under an inverted phase contrast fluorescence microscope (*Olympus, Japan*) after mounted by neutral resins and recorded the growth and damage of hippocampal neurons.

### Statistical analysis

Quantitative data were presented as mean ± standard deviation (SD) from at least three independent experiments. All data were processed and analyzed with SPSS 17.0 (SPSS Inc. Chicago, US). For the content of PGE2, statistical significance was determined by Student's *t* test for pairwise comparisons. For RT-PCR, WB, LDH and MTT data, statistical significance was determined by one-way analysis of variance (ANOVA) with Dunnett's multiple comparisons. *p* < 0.05 was regarded as statistically significant.
